# Editorial: Recording and modulating neural activity in neurodegenerative diseases: Pathophysiological and therapeutic implications

**DOI:** 10.3389/fnhum.2023.1138382

**Published:** 2023-01-24

**Authors:** Andrea Guerra, Gerd Tinkhauser, Alberto Benussi, Tommaso Bocci

**Affiliations:** ^1^Department of Human Neurosciences, Sapienza University of Rome, Rome, Italy; ^2^IRCCS Neuromed, Pozzilli, Isernia, Italy; ^3^Department of Neurology, Bern University Hospital and University of Bern, Bern, Switzerland; ^4^Neurology Unit, Department of Clinical and Experimental Sciences, University of Brescia, Brescia, Italy; ^5^Neurology Unit, Department of Neurological and Vision Sciences, ASST Spedali Civili, Brescia, Italy; ^6^Clinical Neurology Unit, “Azienda Socio-Sanitaria Territoriale Santi Paolo E Carlo” and Department of Health Sciences, University of Milan, Milan, Italy; ^7^“Aldo Ravelli” Center for Neurotechnology and Experimental Brain Therapeutics, Department of Health Sciences, University of Milan, Milan, Italy

**Keywords:** oscillations, deep brain stimulation, Parkinson's disease, beta rhythm, neuromodulation

Neurodegenerative diseases encompass different conditions characterized by progressive degeneration of neurons and networks and accumulation of misfolded proteins in the brain. Recent research employing invasive and non-invasive neurophysiological techniques provided new insights into pathological mechanisms responsible for symptom development. This set the foundation for the translation of advanced therapeutic neuromodulation strategies that hold promise to optimize symptom control and potentially modify disease course in the future. In this Research Topic, we aimed to investigate the role of specific neurophysiological abnormalities in neurodegenerative diseases that serve as electrophysiological target for invasive and non-invasive neuromodulation techniques, such as deep brain stimulation (DBS; Tinkhauser et al., 2017; Bocci et al., 2021) and transcranial alternating current stimulation (tACS; Benussi et al., 2022; Guerra et al., 2022). Parkinson's disease (PD) is an exemplary condition for targeting brain oscillatory activities for therapeutic purposes. Indeed, PD can be considered an oscillopathy, as abnormal oscillations at specific frequency bands in the basal ganglia-thalamo-cortical network play a relevant role in motor symptoms pathophysiology (Oswal et al., 2013). Not only the suppression of exaggerated oscillatory beta activity using DBS (Tinkhauser et al., 2017), but also driving the pro-kinetic high-gamma rhythm at the cortical level using non-invasive tACS may attenuate bradykinesia (Guerra et al., 2022).

Today, DBS of both the subthalamic nucleus (STN) and globus pallidus interna (GPi) are established treatments for PD (Krack et al., 2019). While some controversies on the optimal DBS target selection is still ongoing, even multi or asymmetric targeting can be considered for specific indications. This was demonstrated for the patient reported by Tian et al.. The authors reported the unique case of a 79-year-old male with left limb tremor and right limb stiffness who had a metallic bullet in his brain. Because the bullet was localized on the left STN electrode trajectory, he underwent DBS of the left GPi and right STN. Moreover, due to the bullet he could not undergo pre-operative planning MRI and electrode placement was assured by using CT and intraoperative microelectrode recordings. The patient's symptoms were successfully managed with no discomfort, suggesting that the asymmetric targeting protocol was safe and effective in this particular case.

Abnormally enhanced synchronization in the beta band within the basal ganglia-thalamo-cortical network is a well-established neurophysiological biomarker in PD for indexing bradykinesia and rigidity (Oswal et al., 2013). The improvement of motor symptoms following the suppression of beta activity using DBS or dopaminergic medication, supported the development of closed-loop adaptive DBS (Tinkhauser et al., 2017; Bocci et al., 2021). Evidence is accruing that frequency ranges within the beta band should further be differentiated in the context of brain network characteristics and utility as symptom biomarker. Chen et al. contributed to this article collection, elaborating this Research Topic. They evaluated STN local field potentials (LFP) power in various frequency bands in PD patients. Interestingly, it was found that the power in the high-beta range (20–35 Hz) was positively correlated with the DBS-induced bradykinesia and rigidity improvement and indicated the closest proximity to the active contact used for stimulation. These findings add to the discussion on a more differentiated biomarker selection for LFP-guided DBS programming and adaptive DBS.

The appearance of a non-infectious, transient oedema around the implanted nucleus after DBS implantation is almost a rule and explains the so postoperative clinical stun effect. But what is its impact on the signal quality of STN LFP? Prenassi et al. investigated whether the presence of post-surgery oedema affects the quality of LFP recordings in PD patients undergoing STN-DBS. They integrated the volumetric and spatial information gathered through post-surgery MRI and the LFP signals recorded through the implanted electrodes and found a significant inverse correlation between the left-side LFP in the low-beta band and the oedema volume. While further studies with LFP measurements at different postoperative time points are necessary, the present data already indicate that post-surgery oedema may affect the LFP quality and should be taken in consideration for future LFP-guided optimization of DBS in the acute postoperative phase.

DBS not only improves motor control, but it can also impact on non-motor domains, such as cognition. A retrospective study by Xie et al. assessed the cognitive changes after STN-DBS in 126 PD patients sub-grouped in those with and without mild cognitive impairment (MCI). The authors found that both groups declined cognitively, with an apparently greater cognitive decline in the group with normal baseline cognition. However, a higher proportion of patients progressing to PD with dementia was observed in the MCI group. Our article Research Topic also entails a systematic review by Rački et al. with the goal to gather more generalizable results on this Research Topic. This work includes 67 studies encompassing a total number of 3,677 PD patients implanted in different targets. Data showed that cognitive performance can decline in PD patients and, even if mild in most cases, deficits may impact daily quality of life. The cognitive domain most frequently affected was semantic or phonemic fluency in STN-DBS patients. Across several studies, lower cognitive baseline scores were predictors for a worse outcome in short and long term, which again emphasizes the importance of accurate preoperative clinical assessment.

Overall, the studies published in our Research Topic contribute to the translation of neurophysiology-inspired neuromodulation, ranging from assisted DBS programming to fully automatized adaptive closed-loop DBS. The detailed pre and postoperative clinical, psychological, and neurophysiological assessments will be the backbone for optimal candidate selection and application of these future precision medicine tools.

## Author contributions

AG wrote the Editorial. GT, AB, and TB revised the final version of the paper. All authors listed have made a substantial, direct, and intellectual contribution to the work and approved it for publication.
